# Direct laser carbonization of parylene-C toward microelectrodes for *in vivo* action potential detection

**DOI:** 10.3389/fnins.2026.1893619

**Published:** 2026-08-25

**Authors:** Virgil Christian G. Castillo, Yasumi Ohta, Yoshinori Sunaga, Kuang-Chih Tso, Jun Ohta

**Affiliations:** Strategic Initiative for Research and Innovation, Nara Institute of Science and Technology, Nara, Japan

**Keywords:** action potentials, carbon microelectrodes, electrophysiology, laser carbonization, neural interfaces, red nucleus

## Abstract

Integrating recording electrodes into optical imaging devices presents significant fabrication and material challenges, particularly for lightweight platforms used in freely moving applications. Here, we report an optimized laser carbonization process for the direct fabrication of high-performance carbon microelectrodes compatible with on-chip imaging platforms. We systematically investigated the influence of laser power and repetition rate on the carbonization of parylene-C to maximize graphitization while avoiding ablation. Raman spectroscopy confirmed the formation of graphitic carbon, with the lowest average D-to-G band intensity ratio (I_*D*_/I_*G*_ = 0.63) obtained at 303.86 μW. However, this laser condition resulted in excessive material loss from ablation. The optimal balance between carbon quality and material retention was achieved at 212.69 μW and 30 Hz. Using these parameters, we fabricated implantable carbon microelectrode probes of different sizes (100 × 100, 20 × 20, and 10 × 10 μm^2^) on flexible polyimide substrates. The impedances of these electrodes were 12.10 ± 0.22 kΩ, 596.38 ± 84.86 kΩ, and 8.969 ± 0.991 MΩ for 100 × 100, 20 × 20, and 10 × 10 μm^2^ laser carbonized electrodes, respectively. While larger electrodes offered lower impedance, spontaneous single-unit action potentials were only detectable using the 20 × 20 μm^2^ electrodes, which provided the necessary spatial selectivity to isolate individual neurons. These results establish a maskless fabrication process for high-quality, flexible carbon microelectrodes and demonstrate their suitability for electrophysiological recording.

## 1 Introduction

Neuroscience is built upon, and continually advanced by, the technologies used to probe neural activity. Among these technologies, electrophysiology has been instrumental to countless discoveries in brain function and neural circuitry (Mountcastle, 1957; Häusser, 2000; Tekriwal et al., 2019; Wang et al., 2024). By directly measuring the electrical signals generated by neurons, electrophysiology allows researchers to detect action potentials (AP), the rapid voltage spikes reflecting individual neuronal firing, and local field potentials (LFP), the slower voltage fluctuations reflecting the activity of neuronal populations (Radahmadi et al., 2025; Sinha and Narayanan, 2022). Electrophysiology offers excellent temporal resolution and sensitivity, and recent advances in high-density recording technologies, including silicon probes (Trautmann et al., 2025; Steinmetz et al., 2021; Jun et al., 2017) and flexible electrode arrays (Wei et al., 2024; Lee et al., 2023; Seo et al., 2023; Yasar et al., 2024) have significantly increased the number of neurons that can be recorded simultaneously. However, sampling is spatially sparse and limited to neurons proximal to the recording electrodes, representing only a fraction of neurons within large neural networks. In contrast, fluorescence imaging enables dense spatial sampling across neural populations and can be genetically targeted to specific cell types. These capabilities have driven major advances in our understanding of population-level neural activity (Ghosh et al., 2011; Barbera et al., 2019; Zong et al., 2022; Xue et al., 2023). However, due to the limitation on the reaction kinetics of the fluorescence proteins used, the millisecond resolution necessary for high-fidelity measurements of neuronal spiking cannot be achieved (Zhang et al., 2023). These complementary strengths and weaknesses motivate the combination of electrophysiology with fluorescence imaging to provide a more complete view of brain activity.

Recent advances in multimodal recording systems have addressed several technical challenges of integrating electrophysiology and fluorescence imaging. Recording electrodes have been successfully integrated into GRIN lenses (Cobar et al., 2022) and microprisms (Yang et al., 2024) in head-restrained animal experiments. For freely moving animal experiments, transparent electrode attachments (Wu et al., 2021; Yuan et al., 2025) have been developed for miniaturized fluorescence microscopes but fabricating these electrodes requires specialized microelectromechanical equipment. In contrast, GRINtrodes (McCullough et al., 2022) position electrodes around the lens, preserving the optical path for multiphoton imaging while also simplifying construction. Nevertheless, miniaturized microscopes are still relatively heavy, and reducing device weight is crucial to minimize interference with natural movement (Ohta et al., 2017, 2025).

To address these challenges, we previously reported the integration of electrophysiology with an ultrasmall implantable complementary metal-oxide semiconductor (CMOS) image sensor (Castillo et al., 2024). Through laser carbonization, carbon microelectrodes were directly fabricated on the CMOS device under ambient conditions, eliminating the need for high-temperature fabrication processes typically associated with carbon electrodes (Sharma et al., 2018; Devi et al., 2023), which are incompatible with CMOS circuitry. Using this device, we demonstrated simultaneous recording of fluorescence and LFP signals in the hippocampus.

However, LFPs predominantly reflect the activity of the local neuronal population and, consequently, AP recordings are necessary to resolve single-neuron activity (Buzsáki et al., 2012). Single-unit recordings enable characterization of neuronal firing patterns, spike timing, and population coding dynamics that cannot be obtained from LFP alone (Bean, 2007), providing the single-neuron resolution necessary to leverage the spatial data from our implantable CMOS sensors. In practice, achieving reliable extracellular AP recordings imposes substantially more stringent electrode requirements than LFP recordings due to the smaller signal amplitudes and higher frequency content of spikes (Nelson et al., 2008). In particular, reducing electrode dimensions to isolate single neurons increases electrode impedance and thermal noise, often degrading signal-to-noise ratio (Obien et al., 2015; Viswam et al., 2019). These constraints are especially relevant for laser-carbonized electrodes, whose electrical performance strongly depends on laser processing conditions and the resulting carbon microstructure (Kostecki et al., 2002; Ruan et al., 2018; Iurchenkova et al., 2025).

Consequently, *in vivo* neural interface applications of laser-carbonized electrodes have largely been confined to electrochemical neurotransmitter detection (Zhao et al., 2025), where low impedance at kilohertz frequencies is less critical, and electrocorticography (Lu et al., 2016; Liao et al., 2026), where sub-cellular electrode dimensions are not required. While action potentials have been successfully recorded *in vitro* (Del Duca et al., 2025), the suitability of laser-carbonized electrodes for *in vivo* extracellular action potential recording has rarely been demonstrated, with successful examples reported only for platinum black-functionalized electrodes (Kim et al., 2025, 2026).

In this work, we optimized laser-carbonized microelectrodes for extracellular AP detection and single-unit recording without any surface modifications. To overcome the impedance and thermal noise constraints inherent in decreasing electrode size to achieve single-unit AP recording, we maximized the degree of graphitization (minimize the Raman I_*D*_/I_*G*_ ratio) to ensure sufficiently low electrical impedance. Additionally, we reduced fabrication time by optimizing the laser parameters to operate at a 30 Hz repetition rate. The carbonization process and resulting electrode properties were systematically characterized using Raman spectroscopy, scanning electron microscopy (SEM), and electrochemical impedance spectroscopy (EIS). Using optimized probes, we successfully recorded APs *in vivo* from the mouse red nucleus. To the best of our knowledge, this is the first demonstration of *in vivo* single-unit recording with unfunctionalized laser-carbonized or laser-induced graphene electrodes without relying on metal electroplating.

## 2 Methodology

### 2.1 Laser carbonization

Glass slides were coated with approximately 5 μm of parylene-C via chemical vapor deposition (Labcoter^®^ 2, Specialty Coating Systems). The parylene-C-coated slides were then irradiated with a 266 nm Nd:YAG laser (Callisto VL-C30RS-GV, TNS Systems) with a pulse width of 4 ns and a maximum pulse energy of 10 mJ. Square-shaped electrodes with spot sizes of 10 × 10, 20 × 20, and 100 × 100 μm were patterned directly using the system’s integrated adjustable slit. The laser was operated at power settings ranging from 7.5% to 20.0% and repetition rates of 10, 20, and 30 Hz. Average laser power measurements were performed with an optical power meter (PM101A, Thorlabs) equipped with a thermal sensor head (S405C, Thorlabs).

### 2.2 Materials characterization

Raman spectroscopy (NRS-4100, JASCO) was used to evaluate graphitic carbon formation in the laser-carbonized films. Measurements were performed using a 532 nm excitation laser and a 100 × objective lens, with two accumulations at 300-s exposure. Surface morphology was examined using a scanning electron microscope (SU6600, Hitachi).

### 2.3 Electrochemical characterization

For electrochemical characterization and *in vivo* electrophysiological experiments, approximately 5 μm of parylene-C was coated on custom-made implantable polyimide probes with a 100 × 100 μm^2^ gold electrode. Optimal laser carbonization parameters were used for these carbonizations which was 12.5% power setting at 30 Hz repetition rate for 12,000 shots.

Electrochemical characterizations by electrochemical impedance spectroscopy (EIS) were performed using a potentiostat (PGSTAT 204, Metrohm Japan Ltd., Japan) and a frequency response analyzer (Metrohm Japan Ltd., Japan). Measurements were performed by applying a sinusoidal voltage of 10 mV amplitude over a frequency range of 10^2^–10^6^ Hz in 0.01 M phosphate-buffered saline solution (PBS; FUJIFILM Wako Pure Chemical Corporation, Japan). The laser-carbonized electrode served as the working electrode, a platinum plate (2 × 2 cm) was used as the counter electrode, and an Ag|AgCl electrode (D.6.0733.100 J, International Chemistry Co., Ltd., Japan) was used as the reference electrode.

### 2.4 Electrophysiological recordings

All animal experiments were conducted according to the guidelines provided by the Nara Institute of Science and Technology (NAIST) Animal Committee. The animals used in these experiments were wild type mice [strain C57BL/6JJmsSlc (Japan SLC, Inc, Japan)] maintained in the NAIST Animal Facility with ad libitum access to food and water under a 12-h light/dark cycle.

*In vivo* electrophysiological recordings of the red nucleus in anesthetized mice were conducted to demonstrate detection of APs with the laser-carbonized electrodes. Mice were anesthetized with an intraperitoneal injection of urethane (1.5 g/kg) and then head-fixed to a stereotaxic apparatus. Throughout the surgical procedure, the mice’s body temperature was maintained with a heating pad. Following midline scalp incision, two burr holes were drilled for implanting a recording electrode and for securing a grounding screw. After securing the screw to the skull, either a laser-carbonized or a tungsten electrode was slowly lowered into the red nucleus (−3.0 to −3.2 mm anteroposterior; −0.4 to −0.6 mm mediolateral; 3.0 to 4.0 mm dorsoventral relative to bregma) using a motorized micromanipulator.

The electrode was connected to a preamplifier (SH-MED8, Alpha MED Scientific Inc., Japan) with a 10× gain, which was then connected to a main amplifier (SU-MED8, Alpha MED Scientific Inc., Japan) with a 100× gain. The main amplifier had a 60 Hz notch filter and a 100–5,000 Hz band-pass filter. Signals were digitized using a Micro1401 mkII interface (Cambridge Electronic Design Ltd., United Kingdom) at a sampling rate of 25 kHz with 24-bit resolution.

Electrophysiological recordings were monitored in real time for APs using an audio monitor (Model 3300, A-M Systems, United states). Each putative neuron was recorded for approximately 3 min.

### 2.5 Data analysis

All data processing was conducted using Python 3.12.13 with NumPy 2.4.6 (Harris et al., 2020) within JupyterLab 4.6.0. All visualizations were generated with Matplotlib 3.11.0 (Hunter, 2007). Morphological image processing for determining the percentage of retained carbon was performed using scikit-image 0.26.0 (van der Walt et al., 2014). Spike sorting based on principal component analysis and Gaussian mixture model clustering was carried out with scikit-learn 1.9.0 (Pedregosa et al., 2011).

To quantify carbonization yield, carbonized regions were extracted from high-resolution reflectance micrographs to generate binary masks. Carbonization yield was calculated as the ratio of carbon pixels (*N*_*carbon*_) to the total number of pixels within the laser-irradiated region (*N*_*total*_):


$$\mathrm{Carbonization}\,\mathrm{Yield}=\frac{N_{\text{carbon}}}{N_{\text{total}}}\times 100$$

Specifically, high-resolution reflectance micrographs were imported into Python using the Pillow library and then converted to 8-bit grayscale arrays for pixel-level analysis. Carbonized regions were segmented by converting grayscale images to binary masks using Niblack adaptive local thresholding (block size = 601 pixels). Local thresholding was selected over global methods to compensate for spatial variations in illumination and background intensity across the image field, ensuring consistent segmentation accuracy.

The resulting binary masks were further refined using a sequence of morphological operations to remove speckle noise and improve regional continuity. Specifically, a binary closing operation (3 × 3 structuring element) was applied to fill small discontinuities within carbonized regions. Subsequent area-based filtering (minimum feature size = 50 pixels) removed small artifacts unlikely to represent true carbonized features. The final binary masks were used to determine *N*_*carbon*_ for carbonization yield analysis.

Raman spectra were collected and processed using *Spectra Manager* Version 2 (JASCO Corporation, Tokyo, Japan). Fluorescence background correction was performed using the *Spectra Analysis* module (Version 2.15.22, Build 1). D and G band deconvolution was conducted using the *Curve Fitting* module (Version 2.06.01, Build 1) to calculate for D-to-G intensity ratio. For spectra visualizations, a Savitzky–Golay filter (window length = 8, polynomial order = 1) was applied to smooth the spectra (Barton et al., 2018).

For electrophysiological recordings, raw extracellular recordings were imported into a custom Python-based analysis pipeline. Recordings were trimmed to 1-min segments to standardize data length across samples and limit computational load during clustering. The recordings were then band-pass filtered (300–5,000 Hz) using a finite impulse response filter with a sharp frequency cutoff, allowing fast attenuation of noise outside the AP frequency range.

Noise levels were then estimated using a robust median absolute deviation (MAD) approach. This approach provides a more stable estimate of baseline noise than traditional standard deviation methods, particularly in the presence of transient spikes or non-stationary noise (Rindskopf and Shiyko, 2010). Noise levels (σ_noise_) were estimated as:


$$\sigma_{\text{noise}}=\frac{\text{median}\,\left(\left|x-\text{median}\,\left(x\right)\right|\right)}{0.6745}$$

Spike detection was then carried out using a sliding window to identify local extrema that exceeded a threshold of 4σ_noise_ (Hall et al., 2021).

For spike sorting, each detected waveform was first projected into a lower-dimensional feature space using principal component analysis (PCA), followed by Gaussian Mixture Model (GMM) clustering. PCA condenses waveform information into a low-dimensional feature space that captures essential spike-shape variability, improving computational efficiency (Rey et al., 2015; Takekawa et al., 2010). The GMM framework models each putative neuron as a probabilistic distribution, accommodating overlap between clusters and better reflecting the natural variability of extracellular signals than hard-assignment methods such as k-means (McCaw et al., 2022).

To refine cluster boundaries and improve classification stability, an iterative template-based update procedure was applied. For each cluster template (defined as the mean waveform of that cluster), Euclidean distances between the template and all detected waveforms were calculated (Franke et al., 2015; Navajas et al., 2014). These distances were then re-clustered with a new GMM (using the same parameters) to identify spikes most similar to the current template. Iterations continued until convergence of cluster assignments, as indicated by identical cluster memberships across consecutive iterations.

Finally, outlier rejection was applied within each cluster to ensure that only representative waveforms were retained. For each waveform, the median and MAD were computed across all waveforms in that cluster. Waveforms exceeding ± 2 × MAD from the median were flagged as outliers and removed from further analysis, leaving only stable and biologically consistent spike templates.

## 3 Results

### 3.1 Effect of laser power and repetition rate on parylene-C carbonization

Laser power and repetition rate are important parameters that directly determine the carbonization temperature in parylene-C, the carbon precursor (Montgomery-Walsh et al., 2021). If the power is too low, parylene-C’s temperature will not rise sufficiently to break molecular bonds, and no carbonization will occur. Conversely, excessive laser power can raise the temperature to levels that cause ablation of parylene-C. Therefore, an optimal combination of laser power and repetition rate is required to achieve carbonization.

**FIGURE 1:**
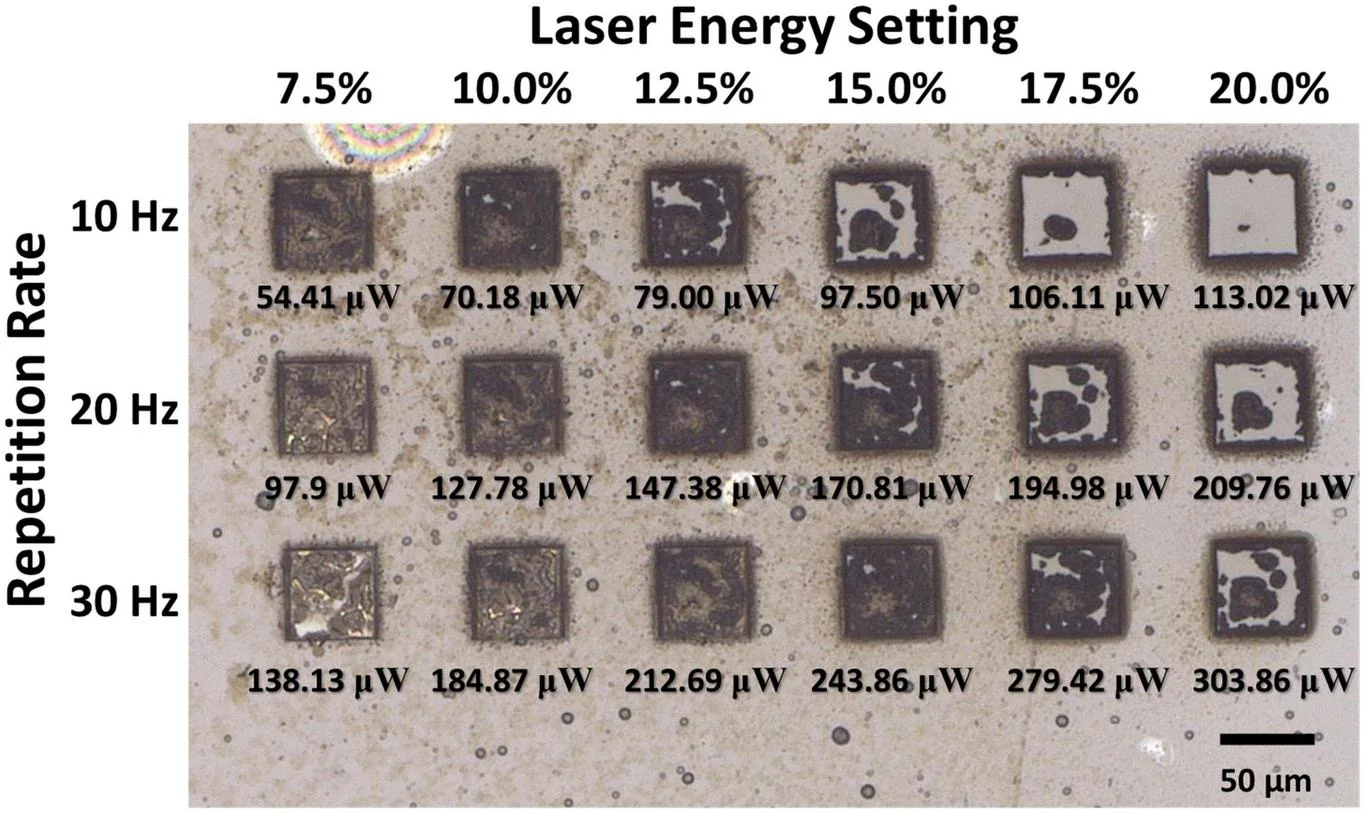
Laser carbonization of parylene-C as a function of laser power and repetition rate at a fixed number of 6,000 laser shots. Each irradiated region is annotated with the corresponding laser power. Parylene-C films exhibit progressive surface darkening with increasing laser power, which eventually leads to ablation. For a given laser power, increasing the repetition rate results in reduced darkening and decreased ablation.

Carbonization of parylene-C can be readily identified by the darkening of the film surface following laser irradiation. Figure 1 shows the evolution of parylene-C carbonization with increasing laser power at different repetition rates. As the laser power increases, the parylene-C film progressively darkens, indicating increasing carbonization. However, beyond certain power levels, ablation of the film is observed, indicating excessive heating. The power thresholds for maximum carbonization and the onset of ablation differ with repetition rate. This effect is evident from the reduced darkening and decreased ablation observed at higher repetition rates for the same laser power.

At each repetition rate, the highest laser power that does not result in ablation (7.5% at 10 Hz, 10.0% at 20 Hz, and 12.5% at 30 Hz) corresponds to the maximum achievable carbonization under those conditions. Among these, carbonization at 12.5% power and 30 Hz is preferred due to the shorter processing time required to fabricate carbon electrodes.

**FIGURE 2:**
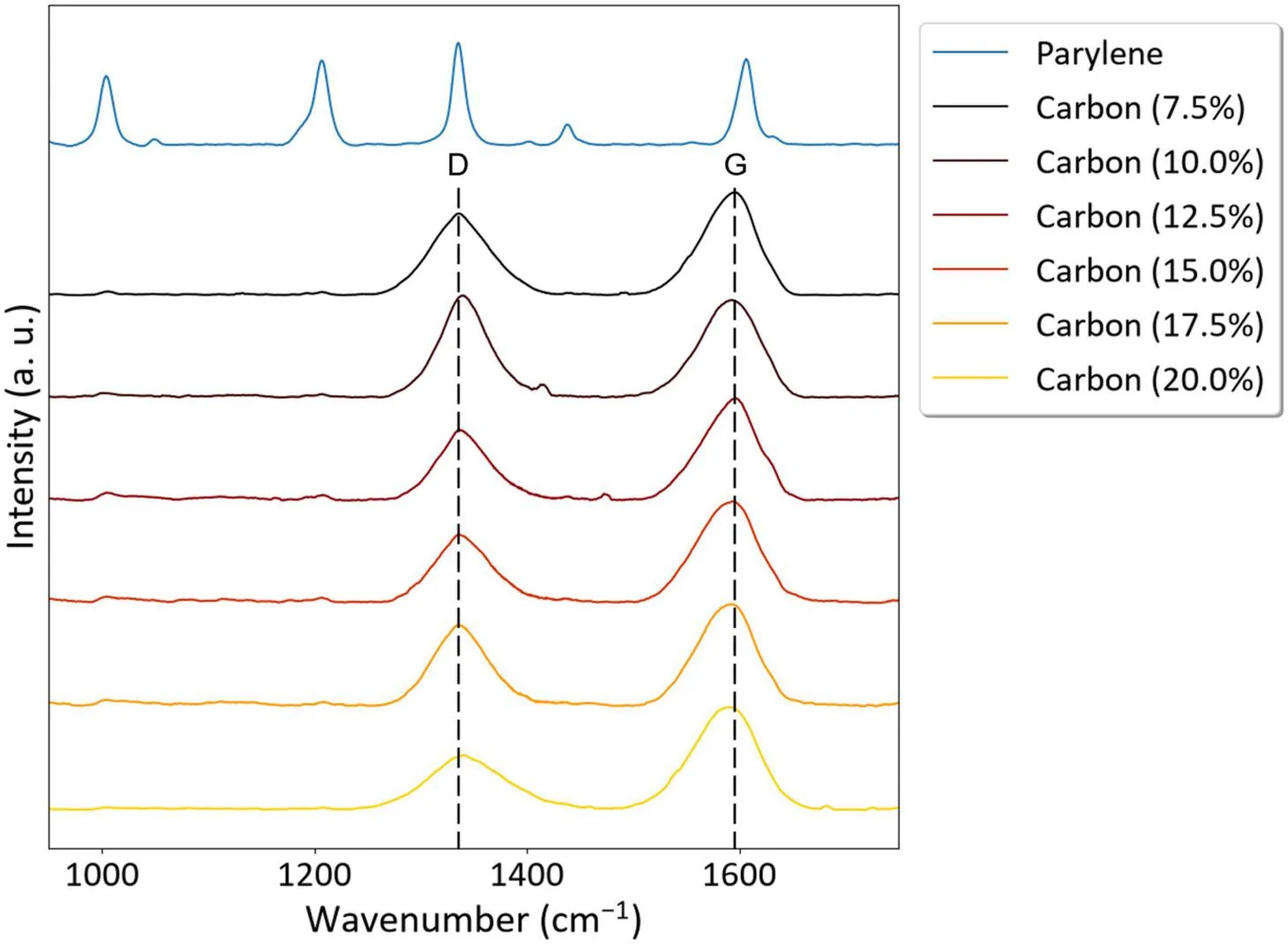
Raman spectra of pristine parylene-C and laser-carbonized parylene-C films at different laser energy settings. Pristine parylene-C exhibited characteristic peaks at 1,005, 1,208, 1,336, and 1,606 cm^−1^, which disappeared after laser carbonization. Carbonized samples showed distinct D (∼1,340 cm^−1^) and G (∼1,590 cm^−1^) bands, confirming the formation of graphitic carbon. The I_*D*_/I_*G*_ ratio decreased with increasing laser power, indicating enhanced graphitic order.

Beyond determining whether carbonization or ablation occurs, laser power and repetition rate also influence the quality of the resulting carbon, which directly affect the material’s properties (Montgomery-Walsh et al., 2021). To confirm carbonization and evaluate carbon quality, we measured the Raman spectra of pristine parylene-C films and the carbonized samples (Figure 2).

The Raman spectrum of pristine parylene-C exhibited characteristic peaks at approximately 1005.13, 1207.69, 1335.91, and 1606.00 cm^–1^, which are attributed to benzene ring symmetric breathing vibrations, C–H in-plane deformations, C–H bending vibrations, and C–H scissoring, respectively (Mathur and Weir, 1973; Wang et al., 2025). There peaks were absent in the laser-carbonized samples, where two new bands appeared at approximately 1,340 and 1,590 cm^–1^.

These are the characteristics D and G bands of graphitic carbon, confirming the successful carbonization of parylene-C. The G band arises from the sp^2^ C–C bond stretching within graphitic domains, reflecting the presence of an sp^2^-bonded carbon lattice in the material. In contrast, the D band is defect-activated and arises from defect-induced scattering at edges and lattice imperfections within the sp^2^ carbon lattice. The ratio of these two bands, I_*D*_/I_*G*_, is widely used to assess carbon quality (Hussein et al., 2016; Phan et al., 2024; Madito, 2025), with a lower ratio indicating a higher fraction of graphitic carbon and a higher ratio reflecting increased disorder and structural defects.

**FIGURE 3:**
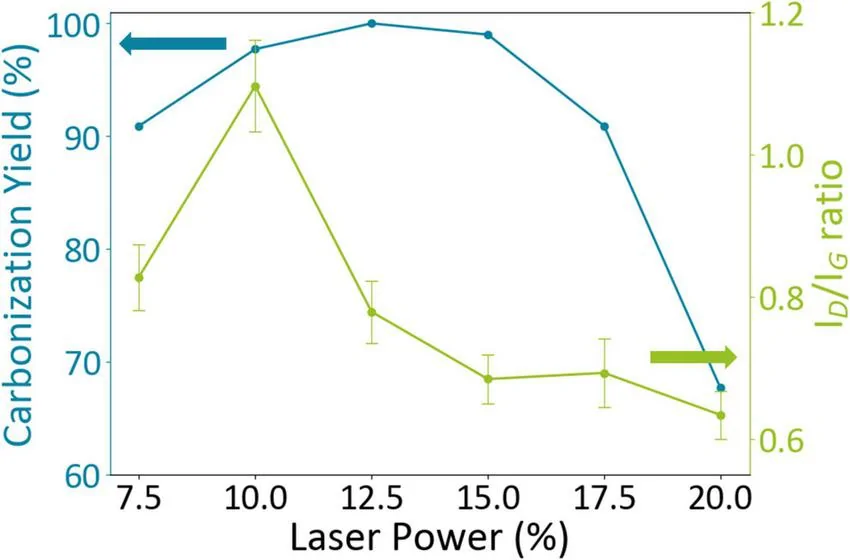
Carbonization yield (left y-axis) and Raman I_*D*_/I_*G*_ ratio (right y-axis) as a function of laser power. The carbonization yield increases with laser power, reaching a maximum at 12.5%, and subsequently decreases at higher powers due to laser-induced ablation. In contrast, the *I*_*D*_/*I*_*G*_ ratio exhibits a peak at 10.0% laser power and decreases with further increases in power, indicating enhanced graphitic ordering at higher energy input. The optimal laser power is 12.5 as it provides the maximum carbonization yield while maintaining a low *I*_*D*_/*I*_*G*_ratio.

In our experiments, carbonization at 20.0% power produced the most graphitic carbon with a mean (±S.E.) I_*D*_/I_*G*_ of 0.63 (±0.03). Similar intensity ratios were observed for 15.0% and 17.5% power at 0.69 (±0.05) and 0.68 (±0.03), respectively. On the other hand, a higher disorder was observed at 7.5%, 10.0%, and 12.5% power with intensity ratios of 0.83 (±0.05), 1.1 (±0.06), and 0.78 (±0.05), respectively. Although the highest carbon quality was achieved at 20.0% power, significant ablation was observed at this setting across all repetition rates (Figure 1), making it unsuitable for electrode fabrication. At 20.0% power and 30 Hz, only 67.67% (Figure 3) of the carbon remained on the irradiated surface. Therefore, considering degree of carbonization, material retention, and carbon quality, the optimal laser parameters were determined to be 12.5% laser power and 30 Hz repetition rate.

### 3.2 Effect of electrode size on impedance

**FIGURE 4:**
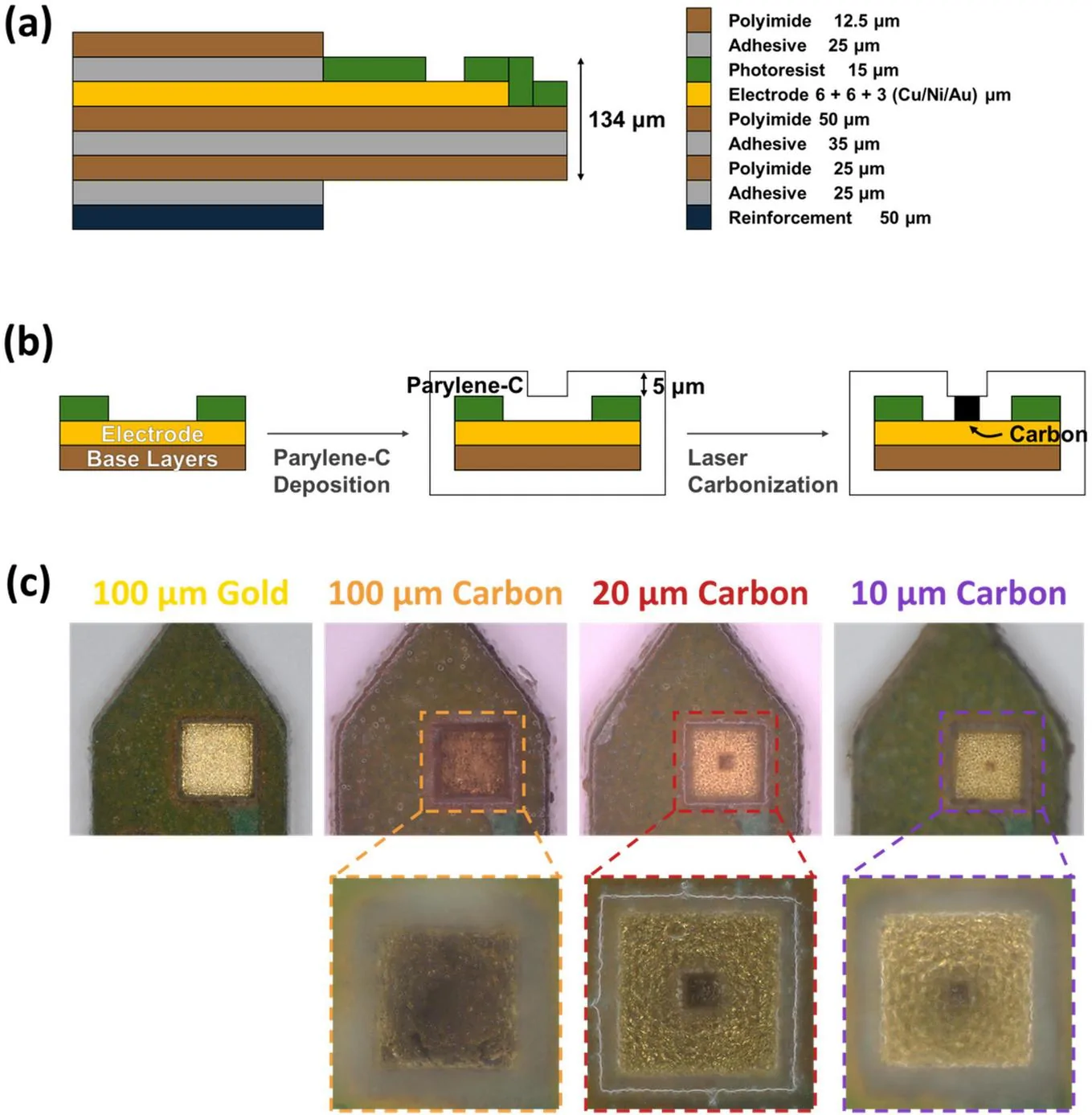
Fabrication and characterization of laser-carbonized electrodes on flexible polyimide probes. **(a)** Cross-sectional schematic of the polyimide probe design. **(b)** Schematic of the laser carbonization workflow, illustrating parylene-C deposition over the gold electrode sites followed by laser carbonization to convert the parylene-C into a conductive carbon film. **(c)** Representative microscope images of a 100 μm gold electrode, 100 μm carbon electrode, 20 μm carbon electrode, and 10 μm carbon electrode, with insets showing magnified views of the carbon electrode sites.

With laser carbonization parameters optimized, we fabricated carbon electrodes of various sizes to investigate how electrode dimension affects *in vivo* AP detection performance. Carbonization was performed on custom-made implantable gold electrode probes with nominal site dimensions of 100 × 100 μm^2^. The probe design is illustrated in Figure 4a. The polyimide substrate provides high flexibility, preventing mechanical failures during brain implantation.

Figure 4b depicts the schematic of the carbonization workflow, in which parylene-C is first deposited over the gold electrode sites, followed by laser carbonization to convert the parylene-C layer into a conductive carbon film. Figure 4c shows representative microscope images of the resulting 100, 20, and 10 μm carbon electrodes alongside an unmodified 100 μm gold electrode, demonstrating the scalability of the laser carbonization process across electrode sizes on the polyimide probe.

**FIGURE 5:**
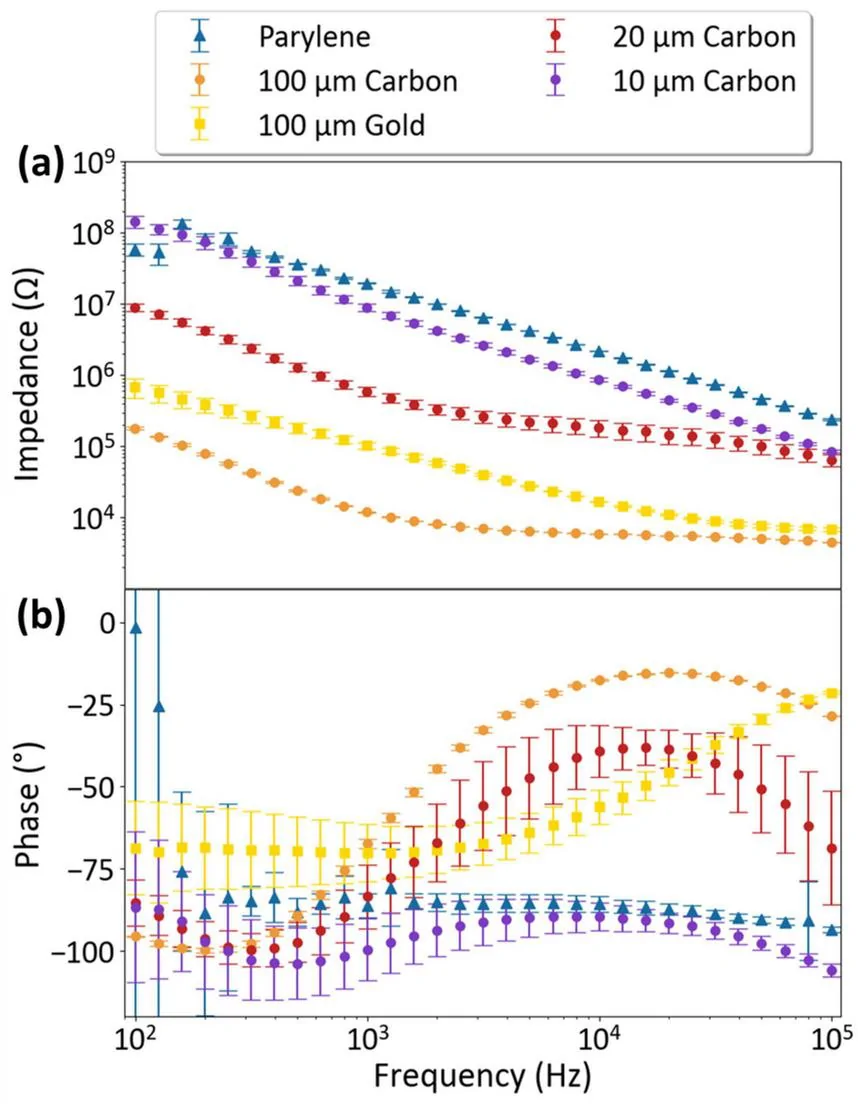
Electrochemical impedance spectroscopy (EIS) Bode plots showing **(a)** impedance magnitude (| Z|) and **(b)** phase angle versus frequency for bare gold, parylene-coated gold, and carbon electrodes. At 1 kHz, 100 μm carbon electrodes (12.10 ± 0.22 kΩ) exhibited lower impedance than gold electrodes (104.85 ± 11.00 kΩ) of the same dimension. Impedance increases to 596.38 ± 84.86 kΩ, and 8.97 ± 0.99 MΩ for 20 and 10 μm electrodes, respectively. Gold electrodes exhibited a single, gradual phase transition characteristic of a planar capacitive interface, whereas 100 and 20 μm carbon electrodes displayed a delayed transition to a more strongly capacitive response due to their microporous structure. The 10 μm carbon electrodes exhibited strongly negative phase angles across the measured frequency range, indicating capacitive behavior similar to insulating parylene.

**FIGURE 6:**
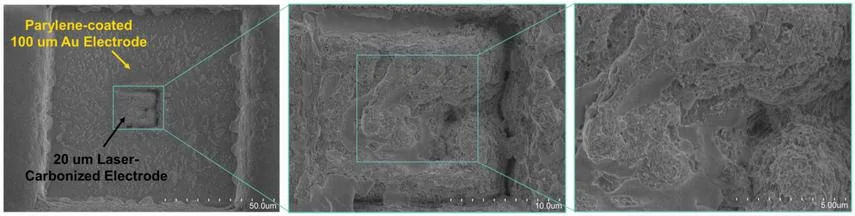
Scanning electron microscopy (SEM) micrographs of 20 μm carbon electrode on 100 μm Au electrode. Higher-magnification images reveal a porous surface morphology with irregular voids and features indicative of increased surface roughness.

To evaluate the electrochemical properties of the electrodes and their suitability for neural recording, we performed EIS. As shown in the impedance Bode plots (Figure 5a), the impedance of 100 μm carbon electrodes at 1 kHz (12.10 ± 0.22 kΩ) were lower than equal-sized gold electrodes (104.85 ± 11.00 kΩ). This difference is attributed to the higher effective surface area of carbon electrodes due to the microporous structure typical of carbonaceous materials, as evidenced by SEM (Figure 6).

As the carbon electrode size decreased, the impedance increased, reaching 596.38 ± 84.86 kΩ for 20 μm electrodes. Further reduction to 10 μm resulted in a sharp increase in impedance to 8.97 ± 0.99 MΩ, approaching that of pristine parylene (19.66 ± 0.41 MΩ).

Phase Bode (Figure 5b) plots were analyzed to gain further insight into the electrodes’ frequency-dependent electrochemical behavior. For all electrodes, the phase becomes increasingly negative with decreasing frequency. This reflects a transition from predominantly resistive behavior at high frequencies, where current is dominated by solution and contact resistances, to capacitive behavior at lower frequencies, where interfacial charge storage becomes significant (Orazem and Tribollet, 2017; Lazanas and Prodromidis, 2023). For equal-sized electrodes, gold exhibits a smooth and gradual decrease in phase from −25° at 1 × 105 to −70° at ∼3 × 10ł Hz. This is consistent with a planar electrode behaving as a single capacitive interface (Schalenbach et al., 2024). In contrast, 100 μm carbon electrodes remain predominantly resistive even at ∼3 × 10^3^ Hz, followed by a more pronounced decrease, reaching −100° at ∼2 × 10^2^ Hz. This delayed phase transition reflects the microporous structure of the carbon electrode, in which a substantial fraction of the electrochemically active surface contributes to charge storage only at lower frequencies. At high frequencies, the outermost regions of the electrode dominate the current response, while slower internal regions progressively contribute as frequency decreases, producing a delayed transition and stronger capacitive behavior at low frequencies (Barcia et al., 2002; Moškon and Gaberšèek, 2021; Lasia, 2025).

For the 10 μm carbon electrodes, a qualitatively different phase response was observed. Strongly negative phase angles (below −75°) were observed across the entire frequency range, similar to those of pristine parylene. This response is primarily capacitive, with negligible resistive current across the electrode surface. For parylene, this is consistent with its insulating nature (Chun et al., 2014), whereas for the 10 μm carbon electrodes, this suggests the material is effectively behaving as an insulator rather than a functional electrode.

Altogether, the EIS results demonstrate the acceptable impedance properties of laser-carbonized electrodes and suggest a microporous structure.

### 3.3 Effect of electrode size on AP detection

To evaluate the effect of electrode size on electrophysiological performance, *in vivo* recordings were performed with 100 and 20 μm electrodes in the red nucleus of mice. Analysis of baseline recordings revealed comparable noise floors across electrode sizes (100 μm: RMS = 5.79 ± 0.74 μV; 20 μm: RMS = 5.49 ± 1.01 μV). This indicates that the recordings are dominated by biological noise rather than the thermal noise of the electrodes (Lempka et al., 2011) and that differences in spike detectability were unlikely to be explained by noise alone. Despite comparable noise floors, APs were only detected with 20 μm electrodes in 4 of 5 recording sessions (*N* = 5 animals, *n* = 4 electrodes), whereas no APs were recorded with 100 μm electrodes in seven sessions (*N* = 7 animals, *n* = 4 electrodes **; *p* = 0.0101, Fisher’s exact test**). This suggests that electrode geometry rather than signal-to-noise ratio is the limiting factor for spike detection. This aligns with the neural electrode design literature on the competing demands of low impedance to enhance signal-to-noise ratio (Lewis et al., 2024; Han et al., 2024), and small geometric area to improve spatial resolution (Viswam et al., 2019; Liang et al., 2022).

**FIGURE 7:**
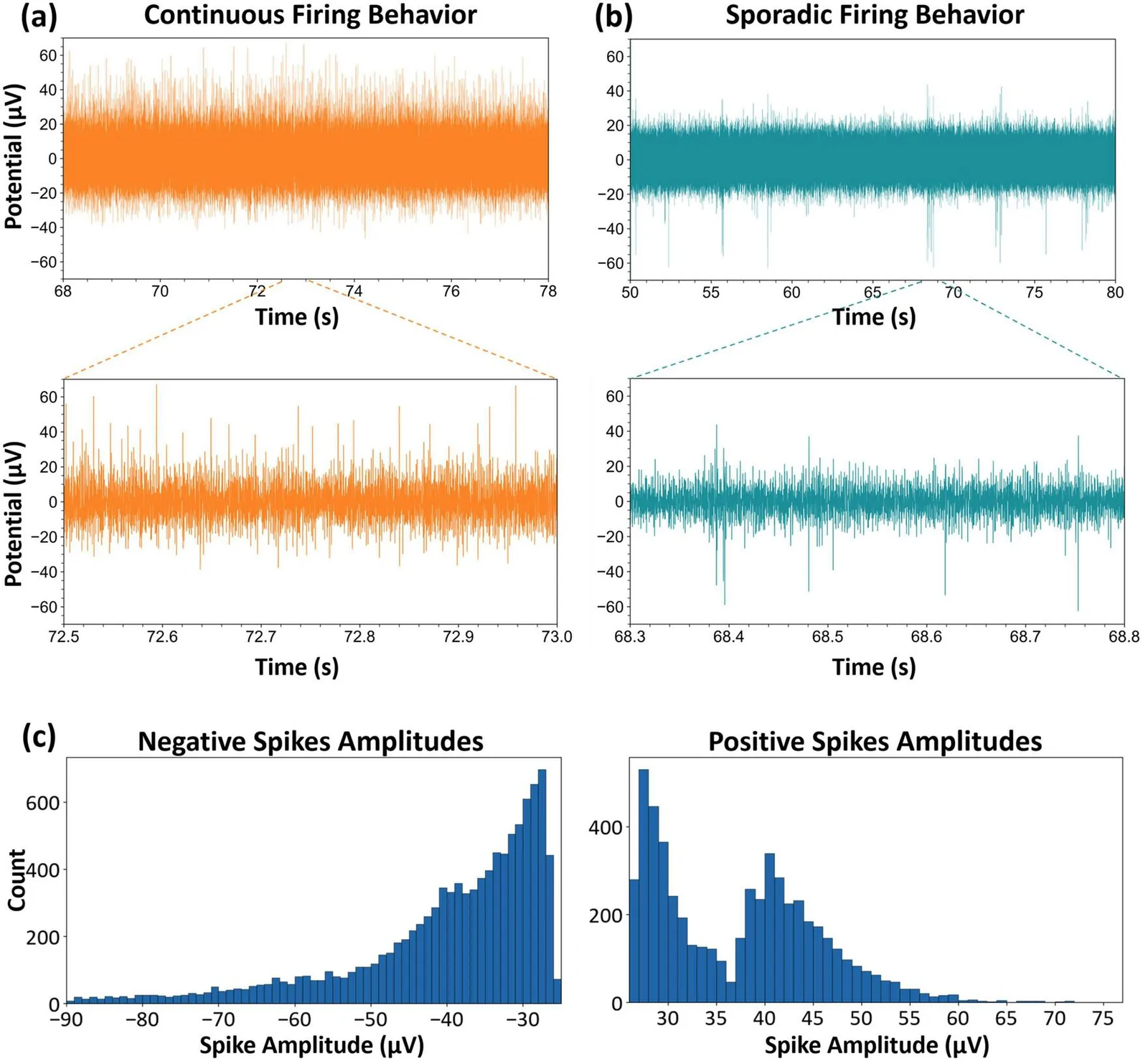
*In vivo* electrophysiological recordings obtained using 20 × 20 μm^2^ laser-carbonized electrodes. **(a,b)** Representative putative neurons exhibiting sustained, continuous firing **(a)** and intermittent, sporadic firing **(b)**. **(c)** Distribution of positive (*n* = 5,550) and negative (*n* = 10,541) spike amplitudes across all recorded sites.

The 20 μm carbon electrodes were used to record spontaneous APs in the red nucleus of mice (*N* = 4 animals, *n* = 4 electrodes). Analysis of the electrophysiological recordings revealed variable firing activity, with some sites exhibiting sustained, continuous firing (Figure 7a) while others displaying intermittent, sporadic bursts (Figure 7b). For negative spikes, the amplitude ranged from −116.55 to −25.38 μV, with a mean (±S.E.) of −40.20 ± 0.13 μV (Figure 7c). On the other hand, the positive spike amplitudes ranged from 25.37 to 76.19 μV, with a mean of 37.31 ± 0.11 μV. Clustering of spike waveforms identified 66 putative neurons, with firing frequencies ranging from 0.17 to 29.02 Hz and a mean frequency of 2.80 ± 0.63 Hz.

**FIGURE 8:**
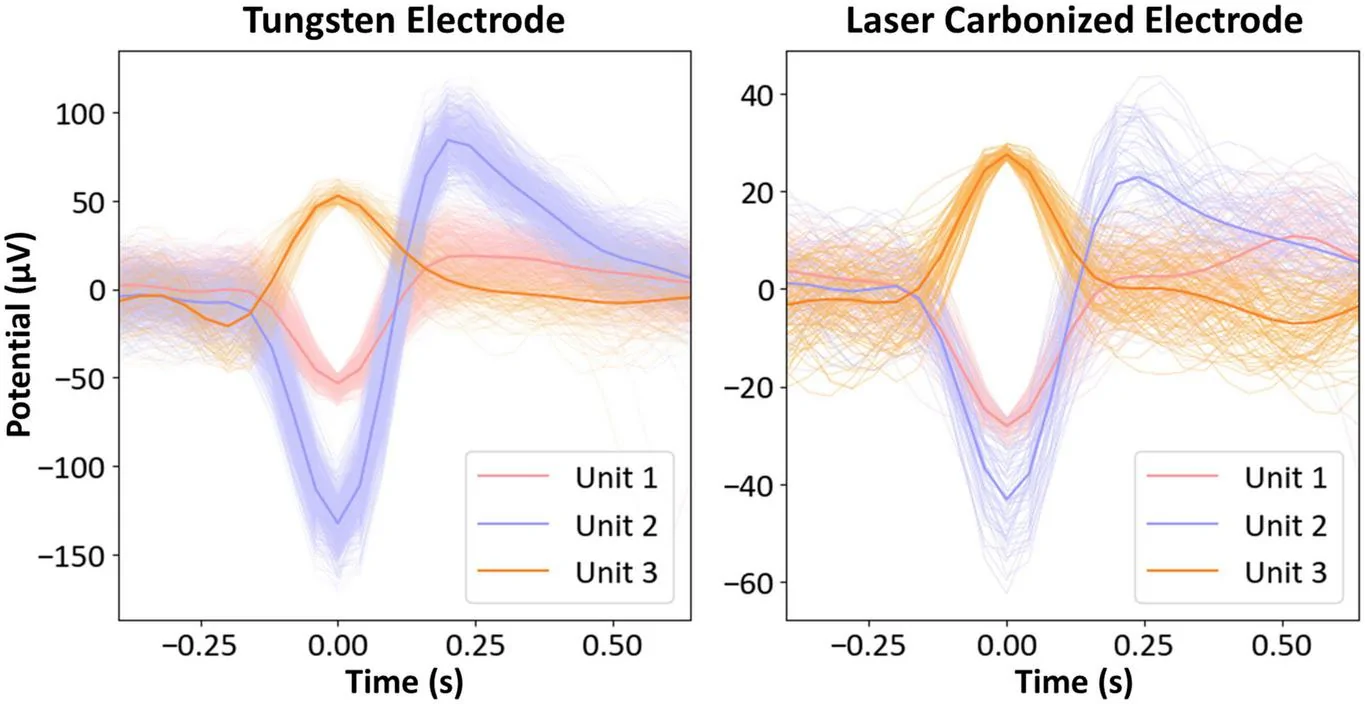
Comparison of waveforms recorded using laser-carbonized and conventional tungsten electrodes. Despite differences in waveform shape due to electrode geometry, spike sorting reveals that laser-carbonized and conventional tungsten electrodes capture comparable single-unit activity.

Electrophysiological recordings with conventional tungsten electrodes were also performed to validate the AP waveforms recorded in Figure 7. It should be noted that the tungsten electrodes differ from the laser-carbonized electrodes in geometry and impedance characteristics, as they represent a distinct electrode type used in conventional applications rather than a matched comparison device. In addition, the acute tissue damage caused by implanting the two electrode types is different, thereby altering the electrode–tissue interface and influencing extracellular recording properties (Ulyanova et al., 2019; Otte et al., 2022). Nonetheless, spike sorting of the recordings obtained in the same brain region revealed single units with comparable waveform characteristics across both electrode types (Figure 8), indicating that the laser-carbonized electrodes can reliably capture the same neuronal activity as conventional tungsten electrodes.

## 4 Discussion

In our previous work, we demonstrated the integration of laser-carbonized electrodes with a CMOS-based imaging platform for recording LFP, establishing the feasibility for monolithic integration of electrophysiological probes into CMOS imaging devices for freely moving animals. Building on that foundation, the present study focuses on the carbon electrode and extends its capabilities to single-unit AP recordings by reducing electrode size and optimizing the carbonization process. This approach allowed us to systematically investigate how electrode geometry and electrochemical properties influence the detection of individual neuronal spikes, providing insights into the trade-offs between impedance, spatial selectivity, and functional recording performance.

The laser carbonization of parylene-C was strongly dependent on both laser power and repetition rate, consistent with previous laser carbonization reports (Correia et al., 2022; Vomero et al., 2018; Chyan et al., 2018). Carbonization requires sufficient energy to break molecular bonds and form graphitic domains, whereas excessive energy leads to ablation and material loss. Raman analysis confirmed that the I_*D*_/I_*G*_ ratio of the resulting carbon films decreased with increasing laser power, indicating higher graphitic content from the increased carbonization temperature (Montgomery-Walsh et al., 2021). However, the highest-quality carbon achieved at 20.0% power resulted in significant ablation, leaving only 64.64% of the laser carbonized film intact. Therefore, the optimal parameters were 12.5% laser power at a 30 Hz repetition rate where a balance between material retention, carbon quality, and fabrication efficiency was achieved.

While these optimized conditions apply specifically to the 5 μm thick parylene-C films used in this study, the optimal laser power is expected to be independent of film thickness. Because parylene exhibits a high absorption coefficient at the 266 nm wavelength (Maggioni et al., 2015), the laser penetration depth is shallow, confining heat generation to a thin layer (Porneala et al., 2016) rather than the bulk material. Therefore, to completely carbonize parylene layers of different thicknesses, the applied laser power should remain constant while the accumulated dose (shot number) is adjusted accordingly.

Following the laser parameter optimization, we investigated how electrode size influences AP detection performance. While 100 μm carbon electrodes exhibited the lowest impedance (12.10 ± 0.22 kΩ), *in vivo* recordings showed that spontaneous single-unit APs were detectable only with 20 μm electrodes, which had an impedance of 596.38 ± 84.86 kΩ. This finding supports previous reports that impedance alone does not dictate AP detection performance (Han et al., 2024; Neto et al., 2018), and factors such as electrode size and spatial selectivity are also critical. However, the 10 μm electrodes exhibited excessively high impedance (8.97 ± 0.99 MΩ), approaching that of uncarbonized parylene. These findings are consistent with the well-established trade-off in neural electrode design, where lower impedance improves signal-to-noise ratio, but small electrodes are required to achieve the spatial resolution necessary to isolate individual neurons (Viswam et al., 2019; Liang et al., 2022). Our results suggest that, for parylene-based laser-carbonized electrodes, an electrode size of 20 μm provides an optimal balance between low impedance and single-unit selectivity.

While the 100 μm gold base utilized in this study inherently limits the spatial density of the current array, this expanded footprint is specific to this initial probe design rather than a fundamental limitation of the carbon electrodes. Future iterations can utilize smaller, matched base dimensions to achieve high spatial density.

*In vivo* recordings from the red nucleus demonstrated that these optimized carbon electrodes could reliably capture APs. The firing patterns observed ranged from sustained, continuous activity to intermittent bursts, and spike clustering identified 66 putative neurons with firing rates from 0.17 to 29.02 Hz. Spike amplitudes were comparable to typical extracellular recordings, with negative spikes averaging −40.20 ± 0.13 μV and positive spikes averaging 37.31 ± 0.11 μV. Comparison with conventional tungsten electrodes revealed that single units detected by laser-carbonized electrodes closely matched those recorded with tungsten electrodes, validating their reliability for functional neural recordings.

This study establishes the acute recording capabilities of these electrodes. Post-recording microscopy showed no carbon detachment or visible wear. However, determining their viability for chronic use requires long-term implantation studies, which will be the focus of future work.

By optimizing laser carbonization parameters and electrode geometry, we successfully fabricated carbon microelectrodes capable of *in vivo* AP detection. These results demonstrate that high-quality carbon electrodes can be reliably produced directly on parylene-coated surfaces under ambient conditions. Future work will focus on applying this approach to implantable miniaturized imaging devices to realize fully integrated, multimodal neural interfaces capable of simultaneous optical imaging and electrical recording at cellular resolution.

## 5 Conclusion

This study establishes a laser carbonization process for fabricating flexible, high-performance carbon microelectrodes directly on parylene-C substrates. Systematic optimization of laser power and repetition rate, identified a critical processing window that maximizes carbonization while minimizing ablation, enabling a maskless fabrication route under ambient conditions without complex cleanroom processing.

We further demonstrate a fundamental trade-off between electrode dimensions and recording performance. While larger electrodes reduced impedance, electrodes with an area of 20 × 20 μm^2^ provided an optimal balance between impedance and spatial selectivity, enabling reliable single-unit AP detection. Comparable AP waveform morphology was observed in red nucleus recordings obtained with laser-carbonized and gold-standard tungsten electrodes, confirming high-fidelity *in vivo* electrophysiological performance.

Importantly, this process is fully compatible with CMOS technology, providing a scalable pathway toward monolithic integration of high-density electrode arrays with implantable imaging platforms. Future implementation on miniaturized implantable imaging devices will enable simultaneous cellular-resolution optical imaging and electrical recording, advancing the development of multimodal neural interfaces for studying neural circuit dynamics in freely moving animals.

## Data Availability

The datasets presented in this study can be found in online repositories. The names of the repository/repositories and accession number(s) can be found below: https://github.com/virgil-castillo/laser-carbonized-parylene-C-microelectrodes.
